# Primary vitreoretinal lymphoma masquerading as refractory uveitis—just go with the flow

**DOI:** 10.1097/j.pbj.0000000000000268

**Published:** 2024-10-14

**Authors:** Leonor Naia, Márcio Tavares, Catarina Ferreira, Sofia Fonseca, Henrique Coelho

**Affiliations:** a Internal Medicine Department, Centro Hospitalar do Baixo Vouga, Aveiro, Portugal; b Hematology Department, Centro Hospitalar de Vila Nova de Gaia/Espinho, Vila Nova de Gaia, Portugal; c Ophthalmology Department, Centro Hospitalar de Vila Nova de Gaia/Espinho, Vila Nova de Gaia, Portugal

## To the Editor:

Primary vitreoretinal lymphoma (PVRL) is a rare and underdiagnosed malignant neoplasm of the central nervous system (CNS) resulting from a monoclonal proliferation of B (>90%) or T cells involving the vitreous, retina, and/or optic nerve.^1-3^ Primary vitreoretinal lymphoma is commonly misdiagnosed because of nonspecific signs, masking other etiologies.^3,4^ An early correct diagnosis is crucial for improving the outcome of this entity.

**Figure 1.**
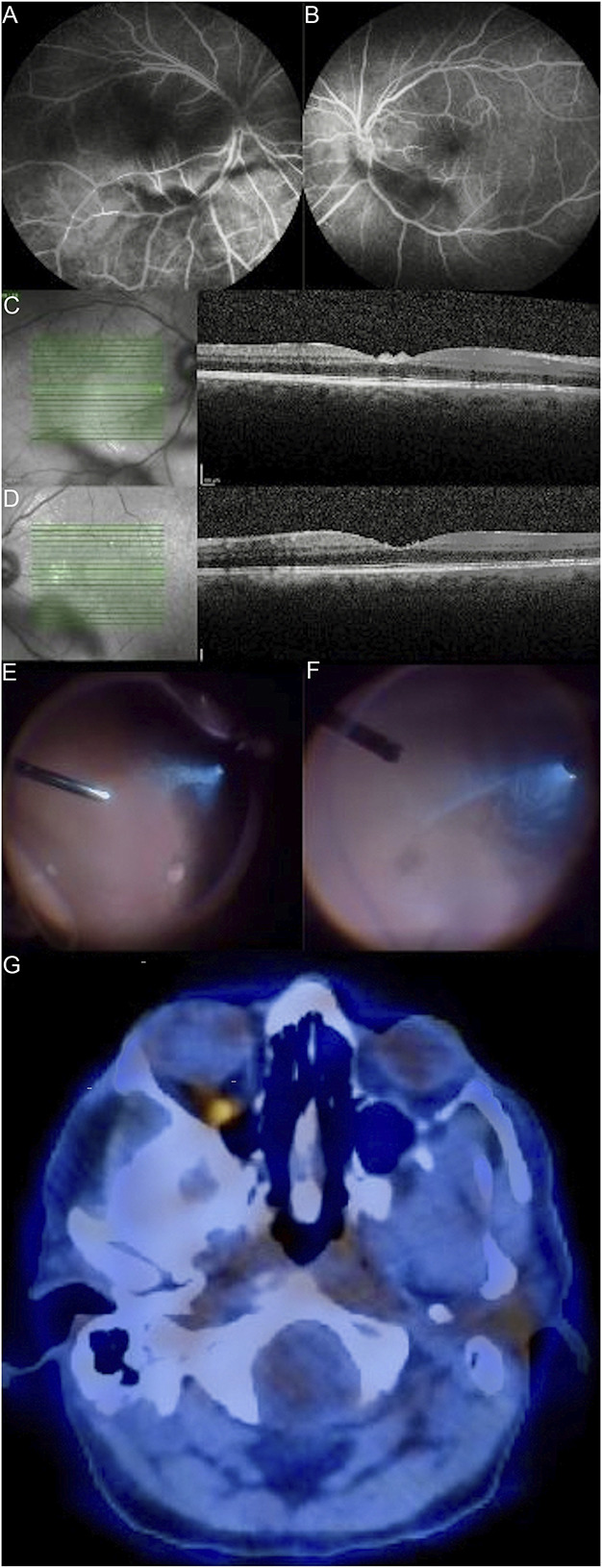
Fluorescein angiography (A and B, respectively) and optical coherence tomography (C and D, respectively) images of the right and left eyes presenting moderate vitritis, with no signs of retinal or choroidal involvement. Pars plana vitrectomy images from each eye (fourth row) showing the appearance of intermediate/large B-cell lymphoma cells in the vitreous cavity, corresponding to homogeneous, large cell sheets that resemble an “aurora borealis” pattern. Positron emission tomography scan (G) revealing asymmetry in the uptake of FDG-F18 in both ocular globes, with increased uptake in the right eye.

A 59-year-old Caucasian man was referred to the Ophthalmology Consultation because of concerns of floaters and blurred vision bilaterally. The patient had a medical history of arterial hypertension, benign prostatic hyperplasia, and bipolar disorder and was medicated with tamsulosin, finasteride, and quetiapine. There was no history of other known systemic or ocular pathology. A complete ophthalmological examination was performed. The best corrected visual acuity of the right and left eyes was 20/63. Anterior segment biomicroscopy and intraocular pressure were normal. Fundus examination revealed moderate bilateral intermediate uveitis, without signs of retinitis. Optical coherence tomography and fluorescein angiography showed intermediate uveitis and signs of mild peripheral vasculitis in the form of periphlebitis (Fig. 1). Systemic autoimmune and infectious workup was negative, and the remaining analytical study was unremarkable. Oral corticosteroid therapy with prednisolone was initiated, with improvement in bilateral best corrected visual acuity and resolution of vitritis. One month later, the patient presented with a maniac episode secondary to steroid therapy. Severe intermediate uveitis relapsed on prednisolone discontinuation.

In the presence of bilateral vitritis of unknown etiology, a diagnostic 23-gauge pars plana vitrectomy with collection of vitreous samples from the left eye was performed. Flow cytometry revealed an intermediate/large monoclonal B-cell population positive for CD19, CD20, CD45, BCL-2, and light-chain restriction for kappa and negative for CD5, CD10, CD38, CD30, and CD138, consistent with intermediate/large B-cell lymphoma. A positron emission tomography (PET) scan was then acquired, showing asymmetry in the uptake of FDG-F18 in both ocular globes, with increased uptake on the right eye; no other hypermetabolic lesions suggestive of high metabolic-grade lymphoproliferative disease were detected. A diagnostic 23-gauge pars plana vitrectomy in the right eye was also performed, showing involvement by intermediate/large B cells with the same immunophenotype. Lumbar puncture, bone marrow biopsy, and brain and spinal cord magnetic resonance imaging were also performed, ruling out other involvement of CNS or systemic involvement. The diagnosis of primary bilateral vitreoretinal B-cell lymphoma was established.

Induction chemotherapy with rituximab (500 mg/m^2^; day 1), methotrexate (3.5 g/m^2^; day 2), vincristine (1.4 mg/m^2^, day 1), and procarbazine (100 mg/m^2^; days 1 through 7) was initiated (R-MVP). A PET scan was obtained after the forth cycle of chemotherapy without evidence of disease. The best corrected visual acuity was 20/63 in the right eye and 20/40 in the left eye, with no evidence of intermediate uveitis. Considering several episodes of decompensation of the psychiatric disease and the evidence of complete response in the interim PET, it was decided to perform only 5 cycles of systemic chemotherapy. After 24 months of follow-up, the patient remains with no evidence of disease.

Primary vitreoretinal lymphoma presents multiple diagnostic challenges because of its atypical presentation, which may include misleading initial response to steroids. A prompt and accurate diagnosis is essential as 60–90% of patients who initially present with vitreous disease may ultimately develop CNS progression, which represents the main cause of death.^5,6^

In our case, the patient presented with unspecific ocular concerns and findings suggestive of intermediate uveitis. Prednisone was initiated based on a possible inflammatory cause. However, in the evidence of a first relapse in a middle-aged patient without a definitive etiological diagnosis, a vitrectomy was performed and confirmed the diagnosis of lymphoma.

Typically, PVRL affects the adults in their fifth or sixth decade of life.^3^ Ophthalmologic imaging techniques can raise the suspicion of PVRL but do not establish the diagnosis. Gold-standard diagnosis continues to be made through invasive diagnostic examinations, namely vitrectomy. Given the diagnostic difficulty, the eye region with greater tumor involvement should be biopsied. Lymphoma cells are fragile and easily degenerate, so retrieved specimens must be promptly transported to the laboratory to minimize cellular degeneration and to maximize the diagnostic yields.^2,4^ The optimal technique for diagnosis may vary among institutions. Cytologic diagnosis is extremely challenging because of hypocellularity of vitreous fluid samples, frequent reactive inflammatory infiltrate that accompanies the tumor response, and previous steroid treatment.^2,4,6^ Flow cytometry is a very useful tool for a rapid diagnosis of B-cell lymphomas and to discriminate infections and uveitis and should be performed in the first vitreous samples.^6-8^ Cytokine analysis and molecular investigation have been developed to increase diagnostic accuracy but are not widely available and are limited by paucicellular vitreous samples.^4,9^ The next step in the diagnostic approach is to rule out systemic or CNS involvement and determine whether the ocular involvement is unilateral or bilateral, as it has therapeutic implications.^3^ Although asymmetrical presentations are encountered, disease is usually bilateral.^2^

In clinical practice, local and systemic therapeutic options are available. Treatment goals in PVRL are both the control of intraocular disease to restore the patient's vision and the prevention of CNS relapse. Intravitreal injection of chemotherapy and local radiotherapy are effective at clearing tumor cells within the eyes but do not prevent CNS relapse. Systemic chemotherapy with high doses of methotrexate offers the best clinical outcomes, with high remission rates and an increase in survival compared with other treatments.^1-4^ Following a relapse, it may be worth considering intensive consolidation chemotherapy, followed by stem cell transplantation.

Mortality ranges from 9 to 81%, and median survival time ranges from 12 to 35 months. The marked variability in prognosis is partially due to the delay in diagnosis.

The timely diagnosis of PVRL can be challenging because of its masquerading characteristics. When PVRL is suspected, flow cytometry can provide helpful diagnostic information within a short turnaround time for prompt start of therapy. In the absence of available standardized protocols, the choice of adequate treatment must be individualized to each patient and must consider the experience of the center.
